# Cognitive and Anatomical Underpinnings of the Conceptual Knowledge for Common Objects and Familiar People: A Repetitive Transcranial Magnetic Stimulation Study

**DOI:** 10.1371/journal.pone.0064596

**Published:** 2013-05-21

**Authors:** Fabio Campanella, Franco Fabbro, Cosimo Urgesi

**Affiliations:** 1 Neurosurgery Unit, Azienda Ospedaliero-Universitaria ‘Santa Maria della Misericordia’, Udine, Italy; 2 Department of Human Sciences, University of Udine, Udine, Italy; 3 Istituto di Ricovero e Cura a Carattere Scientifico ‘E. Medea’, Polo Regionale Friuli Venezia Giulia, San Vito al Tagliamento, Pordenone, Italy; University of Bologna, Italy

## Abstract

Several studies have addressed the issue of how knowledge of common objects is organized in the brain, whereas the cognitive and anatomical underpinnings of familiar people knowledge have been less explored. Here we applied repetitive transcranial magnetic stimulation (rTMS) over the left and right temporal poles before asking healthy individuals to perform a speeded word-to-picture matching task using familiar people and common objects as stimuli. We manipulated two widely used semantic variables, namely the semantic distance and the familiarity of stimuli, to assess whether the semantic organization of familiar people knowledge is similar to that of common objects. For both objects and faces we reliably found semantic distance and familiarity effects, with less accurate and slower responses for stimulus pairs that were more closely related and less familiar. However, the effects of semantic variables differed across categories, with semantic distance effects larger for objects and familiarity effects larger for faces, suggesting that objects and faces might share a partially comparable organization of their semantic representations. The application of rTMS to the left temporal pole modulated, for both categories, semantic distance, but not familiarity effects, revealing that accessing object and face concepts might rely on overlapping processes within left anterior temporal regions. Crucially, rTMS of the left temporal pole affected only the recognition of pairs of stimuli that could be discriminated at specific levels of categorization (e.g., two kitchen tools or two famous persons), with no effect for discriminations at either superordinate or individual levels. Conversely, rTMS of the right temporal pole induced an overall slowing of reaction times that positively correlated with the visual similarity of the stimuli, suggesting a more perceptual rather than semantic role of the right anterior temporal regions. Results are discussed in the light of current models of face and object semantic representations in the brain.

## Introduction

Semantic dementia is a variant form of the degenerative disease called “fronto-temporal lobar degeneration”, which involves the antero-lateral portions of the temporal lobes bilaterally but more commonly in the left hemisphere [1], [2]. The disease causes a highly selective cognitive deficit characterized by the degradation of conceptual knowledge and semantic representations which results into clinical signs of profound anomia and word comprehension deficits. The existence of such a focal disease suggests the possibility that semantic memory store might be, at least at some levels, localized bilaterally in the anterior temporal regions (temporal poles) [3], [4].

In the less common cases of semantic dementia in which the right hemisphere is more involved than the left, the semantic memory deficit tends to be characterized by a progressive difficulty in recognizing particularly familiar people [5]. However, the semantic nature of this deficit remains largely debated. Indeed, in some cases the deficit seems to be more related to a difficulty in retrieving the name, rather than the conceptual knowledge, of familiar people [6]. Moreover, when the deficit seems not related to the retrieving of the names of familiar people, but to their recognition, the syndrome is often referred to as “associative prosopagnosia” [7], [8] or “progressive prosopagnosia” [5], stressing a possible perceptual nature of the deficit.

On the other hand, from the anatomical point of view, a lack of consensus exists in the literature also on the hemispheric lateralization of the deficit in recognizing familiar people. While many studies associated the deficit to damage in the right temporal lobe [5], [9]–[11], other studies found difficulties in identifying familiar people (particularly in name retrieval) after damage to the left hemisphere [12]–[16]. Functional imaging studies did not shed further light on the debate, with some studies indicating activations related to the identification of familiar people bilaterally in the temporal lobe [17], [18], others indicating activation only in the right hemisphere [19] and others in the left hemisphere [20].

### Dissociated representations of objects and faces

Previous neuroimaging and brain lesion studies have provided evidence that objects and faces may have dissociated perceptual and semantic representations. At the perceptual levels, faces activate selective areas of the anterior and posterior occipito-temporal cortex [21], [22] that are less responsive to other object classes. Thus, faces may be a special category of knowledge, whose perception and recognition involve “special” perceptual and semantic systems that are separate from those involved in the “general” perceptual and semantic representations of other objects. However, in contrast to such domain-specific representation hypothesis, Gauthier and coworkers have proposed that faces are not a special “semantic” category per se, but the specificity of faces would rather stem from the special “processing expertise” in making fine grained discriminations among exemplars of the very same subordinate category (e.g., different individuals).

Other studies [20], [23] suggest that faces and objects may differ in the processes and neural structures necessary for their identification, with a stronger right lateralization of the neural underpinnings of face than object identification. However, these differences may lie at a pre-semantic level and occur before accessing a common left anterior temporal representation, which may be responsible for the semantic representation of unique entities, regardless of their category.

Finally, reports (many of which can be found in an interesting review published by Gainotti in 2007) of patients with selective semantic difficulties in identifying familiar people (simultaneously involving different input modalities) suggest that knowledge about familiar people may be dissociated from that of other semantic categories [5], [13], [24]. According to these studies, person-specific information might be independent from general semantic knowledge, which comprises other types of concepts such as, for example, common objects and animals. However, in some of these studies, the deficit was not exclusively limited to the category of familiar people, but extended also to other semantic categories (especially that of living things). The possibility, therefore, exists that the deficit in recognizing familiar people could be just a “by-product” of a more general semantic memory impairment which affects to a greater extent the more “difficult” (less familiar) semantic categories such as that of familiar people or animals. Indeed semantic memory impairments typically manifest as a frequency/familiarity dependent loss of vocabulary meaning, with less familiar concepts being the first to be affected [25], [26]. On the other hand, a very limited amount of studies have reported a reverse dissociation of selective preservation of the (less familiar) category of familiar people in the presence of a general semantic knowledge impairment [5], [11], supporting more clearly the possibility of a segregation of the anatomical substrates of these two categories.

### Semantic nature of difficulties in familiar face recognition

Neuropsychological investigations of brain lesion patients usually attributed impaired performance in word to picture (or picture to word) matching tasks to damage to semantic representations [25], [27]. The semantic nature of the recognition problem is typically supported by the pattern of the patient's errors when the experimenter manipulates semantic variables, such as the familiarity of the concept (or the frequency of the word associated to it) and the semantic relatedness between the target and the distractor stimuli [26]. Indeed, patients with semantic memory problems typically commit a high number of errors that are modulated by concept familiarity or by semantic relatedness. Furthermore, the relative weight of these two variables seems to depend on the nature of the semantic difficulty. When semantic representations are degraded, errors tend to be predicted by the familiarity/frequency of the target concept, with less familiar concepts being odder to recognize. When, on the other hand, patients show a difficulty in *accessing* concepts that are still retained in the semantic store (semantic access dysphasia), errors tend to be more easily predicted by their semantic relatedness, with stimuli being more difficult to recognize when presented with a semantically related distractor than with an unrelated one [26], [28], [29]. Surprisingly, in access dysphasic patients, familiarity effects are much reduced, if not absent.

In the same field of semantic access difficulties investigations, Crutch and Warrington [16] manipulated the semantic relatedness between the target and the distractor face stimuli when testing a patient affected by semantic access dysphasia (AZ). They showed that famous person knowledge might primarily be organized by occupation. In a series of matching to sample tasks, patient AZ showed, indeed, a worse performance in recognizing a target person when presented with distractor people having the same, rather than different occupations. The fact that patient AZ suffered from a stroke involving the fronto-temporo-parietal regions of the left hemisphere supported, moreover, the notion that knowledge for familiar people may rely upon activity of the left hemisphere. However, AZ was found to show semantic access difficulties also for many other categories such as “countries” and “city names” [30], common inanimate objects [31], living things [32] and even abstract concepts [33]. Therefore, the pattern of AZ's deficits might suggest a generalized semantic memory impairment as a consequence of damage to either a unitary and general left lateralized semantic store or to a general semantic retrieval mechanism [34].

While, however, the study of Crutch and Warrington [16] manipulated the effects of semantic distance in the ability of AZ to recognize famous people, the effects of familiarity have never been manipulated in any study of famous people knowledge. This may be probably due to the difficulty of controlling for the subjective level of familiarity of famous people, which drastically varies across different individuals.

### Aims of the present study

A serious limitation of many neuropsychological investigations of familiar people knowledge is that they rely on data coming from patients affected by degenerative syndromes such as semantic dementia, which lead to a progressive degeneration of the cortical regions of *both* temporal lobes, even in those cases in which the damage is reported as *predominantly* left or right [11], [13]. Therefore, it is difficult to exclude that some aspects of the loss of knowledge in a patient with a predominantly left damage is caused by damage to the contralateral hemisphere. More precise evidence in this regard might come from the use of repetitive transcranial magnetic stimulation (rTMS) to temporarily disrupt neural processing of very circumscribed portions of the cerebral cortex [35]. The present study aims therefore to investigate the cognitive and anatomical underpinnings of familiar people knowledge using rTMS in healthy individuals. In particular, we aimed to clarify:

- Whether the cognitive organization of familiar people knowledge is qualitatively dissociable from that of common object knowledge or whether the two categories share similar cognitive principles of organization of their semantic representations (i.e., whether semantic distance and familiarity have similar effects on the ability to identify objects and famous faces).
- Whether the anatomical underpinnings of familiar people knowledge are segregated from those of common object knowledge; thus, whether both knowledge domains are stored within a unitary left lateralized or bilateral temporal semantic network or whether the knowledge about objects is stored in a left temporal network while the knowledge about faces is mainly stored within a right temporal network.
- Whether any differential role of the left and right temporal poles in recognition tasks is attributable to different principles of organization (categorical for left and perceptual for right temporal poles) regardless of the semantic category.

To these aims, we combined a speeded word-to-picture matching task using common objects as well as famous people faces as stimuli and rTMS of the left and right temporal poles. Within the field of concrete concepts we chose to restrict the stimuli to the only category of common manipulable objects, to contrast with that of famous people, since, in the literature, it has been more consistently associated with a clearer left hemisphere lateralization (also often temporal) [6], [36]–[38]. Conversely, the category of living things has been more consistently associated with a more distributed and bilateral representation [39], [40] and may, thus, be less adept for studying the relative role of left and right temporal lobes in semantic coding of distinct semantic categories with unilateral rTMS.

We manipulated the semantic distances and the familiarity of object and face stimuli and searched for specific rTMS effects in the different conditions of the same semantic task. We assumed that any rTMS interference with *semantic* processing should be modulated by the semantic variables considered (i.e., familiarity and semantic distance). In other words, we expect that any rTMS interference on semantic processing should affect only one of the two levels of semantic distance (close vs. distant) and frequency/familiarity (high vs. low). Conversely, the absence of any rTMS effect or nonspecific effects that are not modulated by any of the semantic variables considered will be taken as a sign of effects at a non-semantic level.

## Methods

### Participants

Twenty volunteers (13 female) gave written informed consent for taking part in the study. One participant did not complete all stimulation conditions because of discomfort associated with rTMS of left temporal pole and was therefore excluded from the study. No other discomfort or adverse effects during rTMS were reported or noticed. Mean age of the participants was 24.93 years (SD = 8.43). All participants reported normal or corrected to normal vision, had no history or familiarity for headache or seizures and were free of any psychiatric or neurological illness, other medical problems or any contraindication for rTMS [41]. A standard handedness inventory [42] revealed that all participant were right handed.

### Ethics

The study was approved by the Ethics Committee of the Scientific Institute (IRCCS) “E. Medea” and the procedures were in accordance with the ethical standards of the 1964 Declaration of Helsinki. All participants gave written informed consent.

### Experimental material preparation and selection

#### Objects

Object stimuli consisted of a set of 40 pictures of common manipulable objects (max 350 pixel of height). They were selected and arranged on the basis of the values of word frequency obtained from COLFIS database of written frequency for Italian words [43]: 20 low frequency and 20 high frequency stimuli were selected. The two groups significantly differed in terms of word frequency (Mann-Whitney U test: *Z* = 5.419; *p*<0.001). Stimuli were common manipulable objects varying in terms of their manipulation and affordance. The stimulus set comprised stimuli that are typically manipulated with the right (e.g. hammer) or left hand only (e.g. watch) or with both hands (e.g. pot) or that are not manipulated with hands (e.g. pacifier). The orientation of the two probes was kept comparable in each array and we avoided any systematic bias in the orientation and affordances of the manipulable objects with respect to the hand used for answering.

#### Faces

Face stimuli consisted of a set of 40 pictures of famous people (275×350 pixels). While measures of word frequency are available for written words in several databases for common objects [43], similar measures for the category of famous people were not available. However, since word frequency is typically positively correlated with measures of concept familiarity [44], [45], an *ad-hoc* set of norms of familiarity was collected and used for this category. A group of 24 independently sampled university students (mean age: 23.4; range: 19–52) was asked to judge how familiar they were with the person depicted on the stimulus picture. A set of pictures of the face of 148 male and female famous people belonging to different occupational fields was preselected and presented in a random sequence to participants on a 15″ 1024×768 laptop screen. For each picture, participants were first asked to report whether they had ever seen the face (i.e., to judge the feeling of familiarity with the person) by pressing one of two keys on the keyboard corresponding to Y or N responses. After this first answer, the name of the person appeared under the picture and they were further asked to judge how familiar the person was to them on a 7-point scale.

The overall mean recognition rate (number of Y answers) was 91% (SD = 7%) across participants. Two participants were removed from the analysis due to excessively low recognition rate (70% and 73%, respectively; cutoff = 76%). All the stimuli that were not recognized by at least 90% of the remaining participants were removed from the stimulus list. This procedure allowed us to reduce the interindividual variability in the knowledge of famous people and to assure, as far as possible, that all the stimuli used in the task were known by all participants. Experimental stimuli were then chosen among the remaining 119 stimuli on the basis of their familiarity: 20 stimuli were selected with low (mean: 5.25; SD = 0.53) and 20 with high (mean: 6.20; SD = 0.27) level of familiarity. The two groups of stimuli significantly differed in familiarity (Mann-Whitney U test: Z = 4.977; p<0.001). In each trial, the two probe face stimuli were largely matched for the depicted emotional expression and contextual information, thus ensuring that matching the target name relied on the recognition of facial identity.

For both categories, the selected stimuli were arranged in 10 groups of 4 stimuli (5 high and 5 low in familiarity), each composed of two pairs of closely related stimuli. The semantic relatedness criterion was contextual/functional for the category of objects (e.g., two kitchen tools and two garden tools; or two writing tools and two office tools) and contextual/occupational for the category of famous people (e.g., two anchor men and two football players; or two movie stars and two politicians). For both categories, the distant pairs were obtained by crossing the stimuli of each pair within each group. For each group, each stimulus appeared two times as target (once with a close distractor and once with a distant one) and two times as distractor. Overall there were 80 trials for each of the two categories (Objects and Faces): 20 high familiarity and semantically related, 20 high familiarity and semantically unrelated, 20 low familiarity and semantically related, and 20 low familiarity and semantically unrelated.

### Experimental procedure

**Figure 1:**
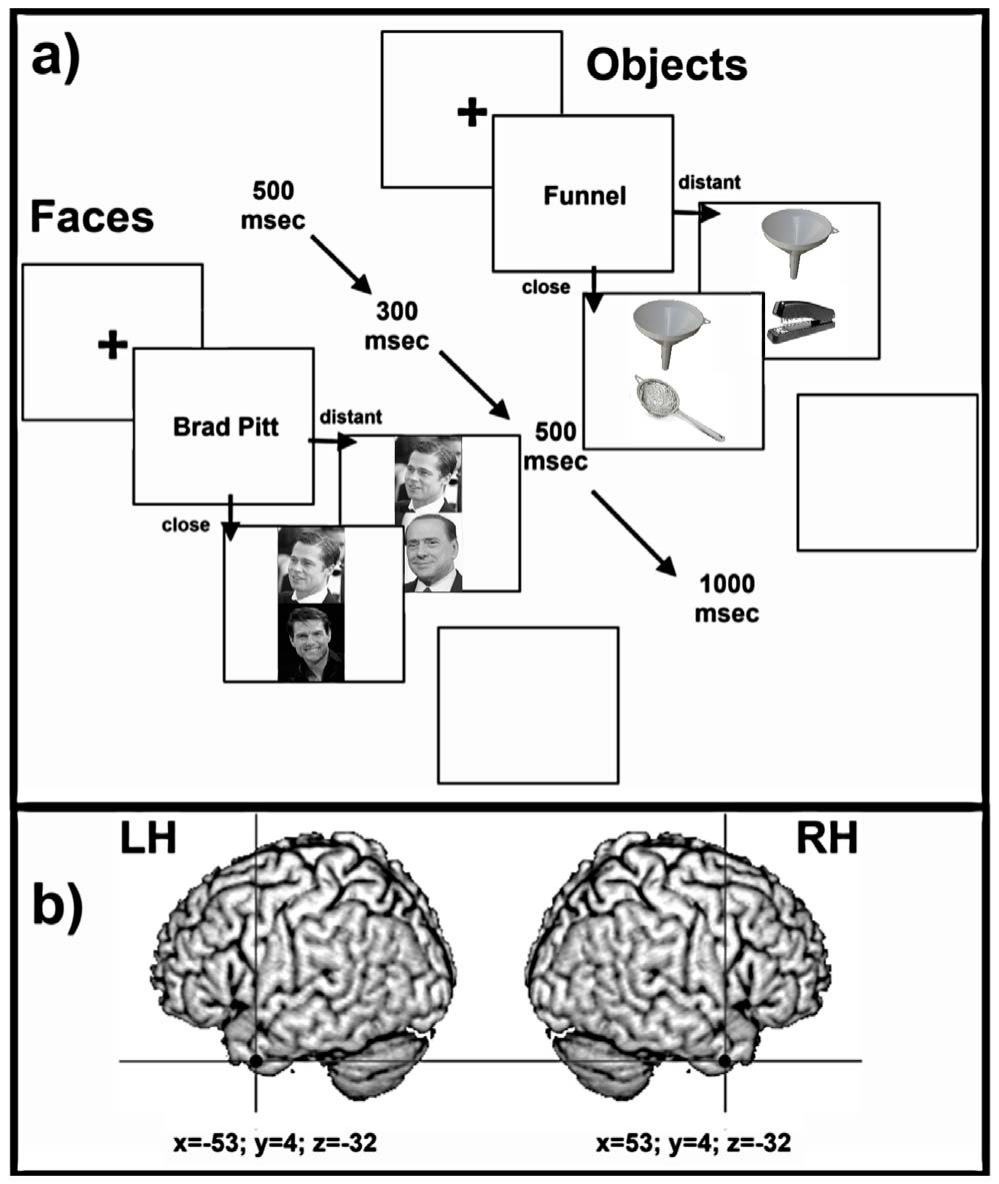
Event Sequence. panel a) event sequence and timing for both experimental conditions (Objects and Faces); panel b) anatomical coordinates of the stimulation sites for both rTMS conditions. The experimental face and object stimuli are replaced in the picture with similar stimuli obtained through an Open Access source: http://commons.wikimedia.org and are usable under CCAL. Licence permissions for each of the pictures can be found at the following links: Brad Pitt picture: adapted from http://commons.wikimedia.org/wiki/File:Angelina_Jolie_Brad_Pitt_Cannes.jpg. Attribution: Georges Biard [CC-BY-SA-3.0 (http://creativecommons.org/licenses/by-sa/3.0)], via Wikimedia Commons. Tom Cruise picture: adapted from http://commons.wikimedia.org/wiki/File:TomCruiseDec08MTV_cropped.jpg. Attribution: MTV Live [CC-BY-SA-2.0 (http://creativecommons.org/licenses/by-sa/2.0), GFDL (http://www.gnu.org/copyleft/fdl.html) or CC-BY-SA-3.0 (http://creativecommons.org/licenses/by-sa/3.0/)], via Wikimedia Commons. Silvio Berlusconi picture: adapted from http://commons.wikimedia.org/wiki/File:Silvio_Berlusconi_%282010%29-modif.png Attribution: Public domain: By www.la-moncloa.es Derivate work: Habib M′henni (Transparency) (www.la-moncloa.es) [Public domain], via Wikimedia Commons. Funnel picture: adapted from http://commons.wikimedia.org/wiki/File:Kitchen_Funnel.jpg. Attribution: By Donovan Govan. [GFDL (http://www.gnu.org/copyleft/fdl.html) or CC-BY-SA-3.0 (http://creativecommons.org/licenses/by-sa/3.0/)], via Wikimedia Commons. Strainer picture: adapted from http://commons.wikimedia.org/wiki/File:Kitchen-Strainer.jpg. Attribution: Public domain: By Evan-Amos (Own work) [Public domain], via Wikimedia Commons. Stapler picture: adapted from http://commons.wikimedia.org/wiki/File:Black_Stapler.jpg. Attribution: By ZooFari (Own work) [CC-BY-SA-3.0 (http://creativecommons.org/licenses/by-sa/3.0)], via Wikimedia Commons.

We used a speeded written word-to-picture matching paradigm. Stimuli were presented on a 1024×768, 15″ laptop pc monitor (refresh frequency, 60 Hz) located at a distance of approximately 57 cm from the participant. Stimuli subtended a 7.3°×9.3° region and were presented on a white background. Stimulus presentation timing and randomization were controlled using E-Prime v.1.2 software (Psychology Software Tools Inc, Pittsburgh, PA). For each trial the procedure was the following (see also Fig.1): a fixation cross remained at the center of the screen for 500 ms, followed by the brief presentation of the target name in the center of the screen for 300 ms. Then, an array of two probe stimuli was presented (one in the upper and one in the lower half of the screen) for 500 ms followed by a blank screen for 1,000 ms. Participants were required to identify the target name by pressing, as fast and accurate as possible, with their right index or middle finger one of two keys on the keyboard, aligned on the vertical line and labeled as 1 and 2, to indicate whether the target name corresponded to the upper or lower probe stimulus. The positions of the matching and non matching probe stimuli were randomized in each trial. The time limit for providing the response was within 1,500 ms after the onset of the probe stimulus array (i.e., before the offset of the blank screen). At the offset of the blank screen, a new trial was presented. Accuracy and reaction times (RTs) were recorded and stored for automatic analysis.

Each participant performed each task (Objects and Faces) in three conditions: a) after the stimulation of the left temporal pole (lTP-rTMS condition); b) after stimulation of the right temporal pole (rTP-rTMS condition) and c) in absence of any stimulation (no-rTMS condition). The three conditions were administered in three different consecutive sessions administered in the same day. To reduce the magnitude of potential learning effects due to task repetition within short period of time, three versions of each task were prepared. Stimuli and procedures were identical, but three different pictures of each stimulus exemplar were used. The order of presentation of the trials was automatically randomized by the software, while the order of presentation of the two categories (Faces or Objects) as well as of the stimulation conditions (lTP, rTP or no-rTMS) and task versions (1, 2, or 3) was counterbalanced across participants. Thus, across participants, the three versions of the task were presented in all the three stimulation conditions.

### Semantic distance, familiarity and visual similarity judgments

After the administration of all the three experimental sessions, all subjects (but one) performed a supplementary rating session in which they were asked to judge using a 7-point Likert-like scale the level of semantic relatedness, familiarity and of visual-perceptual similarity between the stimuli in each pair from the experimental material.

### Stimulation procedure

An off-line rTMS stimulation protocol was adopted: participants performed the behavioural tasks after 15 minutes of low frequency (1 Hz; 900 pulses) rTMS stimulation released over the lTP and rTP and in a no-rTMS condition. RTMS pulses were delivered using a Magstim Rapid stimulator (Magstim Co., Whitland, UK) with a biphasic current waveform, producing a maximum output of 2 T at the coil surface (pulse duration, 250 µs; rise time, 60 µs), which was connected to an eight-shaped air-cooled coil (outer diameter of each wing, 7 cm). Prior to rTMS, the resting motor threshold of the participants was estimated by releasing single magnetic pulses to the optimal scalp position for evoking motor evoked potentials with maximal amplitude from the right first dorsal interosseous muscle (FDI). Electromyographic recordings from the FDI muscle were performed through surface Ag/AgCl cup electrodes (1-cm-diameter) placed in a belly-tendon montage. Responses were amplified, band-pass filtered (20 Hz–2 kHz) and digitized by means of a Viking IV electromyography equipment (Nicolet Biomedical, Madison, WI). The sampling rate of the EMG signal was 20 kHz. A pre-stimulus recording of 80 ms was used to check for the presence of EMG activity before the TMS pulse. The resting motor threshold was defined as the lowest stimulus intensity able to evoke five out of ten motor evoked potentials with an amplitude of at least 50 µV while holding the stimulation coil over the optimal scalp position for the FDI muscle. Resting motor threshold values varied from 40% to 72% (mean  = 55.31%). During rTMS of both lTP and rTP, the stimulator output was set to an intensity of 100% of the individual resting motor threshold.

The coordinates in Talairach of the stimulation sites were x = −53, y = 4, z = −32 for lTP and x = 53, y = 4, z = −32 for rTP and were taken from previous rTMS studies investigating semantic memory representation and targeting the same anatomical locations [46], [47]. These areas were located on each participant's scalp with the SofTaxic Optic - neuronavigation system for TMS (Electro Medical Systems, Bologna, Italy; http://www.softaxic.com). Skull landmarks (nasion, inion, and two preauricular points) and 60 points providing a uniform representation of the scalp were digitized by means of a Polaris Vicra optical tracking system (Northern Digital Inc.). Coordinates in standard space were automatically estimated by the SofTaxic Otpic system from a magnetic resonance imaging-constructed stereotaxic template, which also allowed on-line monitoring of the position of the coil focus over the target positions during stimulation. The coil was placed and securely held tangentially to the scalp by means of a coil holder, with the handle pointing backward and approximately parallel to the temporal gyri. After the rTMS of lTP and rTP or in the no-rTMS condition, participants performed the word-to-picture matching task for both categories (Objects and Faces). Performing the task for both categories had a maximal duration of 10 minutes, thus within the time limit of the estimated effects of temporal pole stimulation on semantic tasks [46], [47]. The interval between two consecutive stimulation conditions was at least 60 min, thus ensuring that any residual effect of rTMS had faded away.

### Data handling

For each category and each combination of frequency/familiarity, individual mean percentages of correct responses and reaction times (RTs) were separately calculated for each rTMS condition (20 trials per cell). Only RTs of correct responses were considered for the analysis. A 2×3×2×2 full-within subjects design was adopted, with category (Objects vs. Faces), rTMS condition (no-rTMS, lTP, and rTP), semantic distance (close vs. distant) and familiarity (high vs. low) as within-subject variables. Table 1 reports the mean accuracy and RTs values in each condition. Accuracy and RTs were entered into separate repeated-measures Analyses of Variance (ANOVAs) and post-hoc comparisons were made by means of the Duncan Test. A significance threshold of p<0.05 was set for all statistical analyses.

**Table 1:** Mean accuracy and reaction times (+/− standard error) for each condition of semantic distance, frequency/familiarity and rTMS stimulation.

ACCURACY					
CATEG	STIM SITE	DISTANCE	FAMILIAR	Mean Acc(%)	+/− SE (%)
Objects	lTP	Close	High Fam	81.5	2.4
			Low Fam	78.1	2.3
		Distant	High Fam	97.6	0.7
			Low Fam	91.5	2.0
	rTP	Close	High Fam	80.9	2.5
			Low Fam	79.7	2.9
		Distant	High Fam	97.1	1.2
			Low Fam	92.9	1.6
	no-rTMS	Close	High Fam	81.2	2.8
			Low Fam	79.7	2.2
		Distant	High Fam	97.9	0.9
			Low Fam	91.0	1.4
Faces	lTP	Close	High Fam	75.3	3.4
			Low Fam	70.4	3.7
		Distant	High Fam	91.4	1.6
			Low Fam	80.3	3.0
	rTP	Close	High Fam	81.3	2.8
			Low Fam	74.4	3.4
		Distant	High Fam	91.8	1.7
			Low Fam	82.2	3.0
	no-rTMS	Close	High Fam	80.5	3.5
			Low Fam	71.3	3.7
		Distant	High Fam	87.4	2.0
			Low Fam	85.4	2.3

REACTION TIMES					
CATEG	STIM SITE	DISTANCE	FAMILIAR	Mean Acc(%)	+/− SE (%)
Objects	lTP	Close	High Fam	489.507	20.908
			Low Fam	464.611	17.210
		Distant	High Fam	417.251	10.403
			Low Fam	448.787	12.101
	rTP	Close	High Fam	485.454	23.209
			Low Fam	473.634	21.374
		Distant	High Fam	427.462	13.945
			Low Fam	463.608	18.265
	no-rTMS	Close	High Fam	463.847	14.189
			Low Fam	456.731	12.877
		Distant	High Fam	413.811	9.925
			Low Fam	442.525	11.588
Faces	lTP	Close	High Fam	498.183	17.485
			Low Fam	502.272	19.547
		Distant	High Fam	494.101	17.267
			Low Fam	510.035	18.639
	rTP	Close	High Fam	516.065	26.331
			Low Fam	520.691	25.306
		Distant	High Fam	497.272	23.184
			Low Fam	507.704	21.948
	no-rTMS	Close	High Fam	497.158	19.537
			Low Fam	504.069	17.392
		Distant	High Fam	490.425	18.300
			Low Fam	493.366	12.786

## Results

### Semantic Distance and Familiarity Ratings:

**Figure 2:**
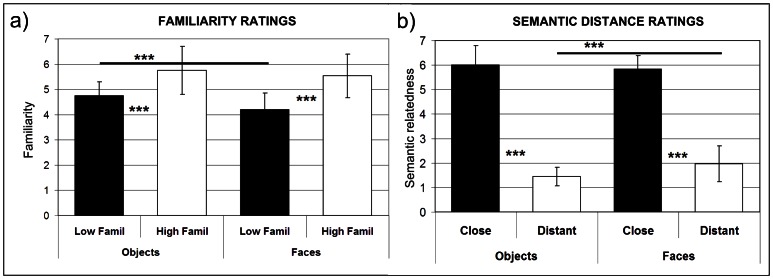
Semantic relatedness and familiarity ratings. Ratings provided by participants at the end of the experimental session. *  = p<0.05; ** = p<0.01; *** = p<0.001.

Figure 2 illustrates the mean semantic distance and familiarity values for the ratings provided by the participants at the end of the experimental session. Two separated Friedman ANOVAs were performed for familiarity and semantic distance, respectively. Post-hoc comparisons were then performed by means of a series of Wilcoxon Matched Pairs tests and p-levels were corrected for multiple comparisons (p = 0.05/6 = 0.008). A general familiarity effect was found across categories (Chi Square_(N = 18, df = 3)_ = 35.413; p<0.001). Familiarity ratings (Fig. 2a) were lower for faces than objects for low (Z = 2.651; p = 0.008) but not high familiarity stimuli (Z = 0.043 p = 0.965). Within each category a largely significant difference separated high and low familiarity items (Z = 3,723; p<0.001 for objects and Z = 2.896; p = 0.004 for faces). Regarding the semantic distance ratings, a large difference separated, for both categories, close from distant pairs (Z = 3,723; p<0,001 for objects and faces). However, while close face and object pairs were rated comparably related (Z = 0.218; p = 0.828), distant faces were found to be slightly (but significantly) more related than distant objects (Z = 3.393; p<0.001).

### Accuracy

The ANOVA on accuracy failed to reveal any rTMS modulation of performance. Indeed, the main effect of rTMS condition and its 2-, 3-, and 4-way interactions with category, semantic distance and familiarity were not significant (all Fs<2.271; p>0.118). A main effect of category (F_(1,18)_ = 18.292; p<0.001; η^2^ = 0.504), with lower accuracy for the category of faces than objects was found. Moreover, there were significant main effects of both semantic distance (F_(1,18)_ = 101.800; p<0.001; η^2^ = 0.850) and familiarity (F_(1,18)_ = 46.966; p<0.001; η^2^ = 0.723), with a lower accuracy for closely related and for low familiarity items.

Both variables interacted significantly with semantic category (Category × Distance: F_(1,18)_ = 15.568; p<0.001; η^2^ = 0.464; Category × Familiarity: F_(1,18)_ = 10.933; p = 0.004; η^2^ = 0.378), suggesting that their influence was different for the two categories. Semantic distance and familiarity effects were largely significant for both categories (all ps<0.001), but semantic distance effects were larger for objects (distant − close difference  = 14.5%) than for faces (distant − close difference  = 10.9%), while familiarity effects were higher for faces (high − low difference  = 7.3%) than for objects (high − low difference  = 3.9%). These results suggest some possible differences between the semantic representations of faces and objects; however, rTMS induced no reliable behavioral changes for any categories at this level of analysis.

### Reaction Times

**Figure 3:**
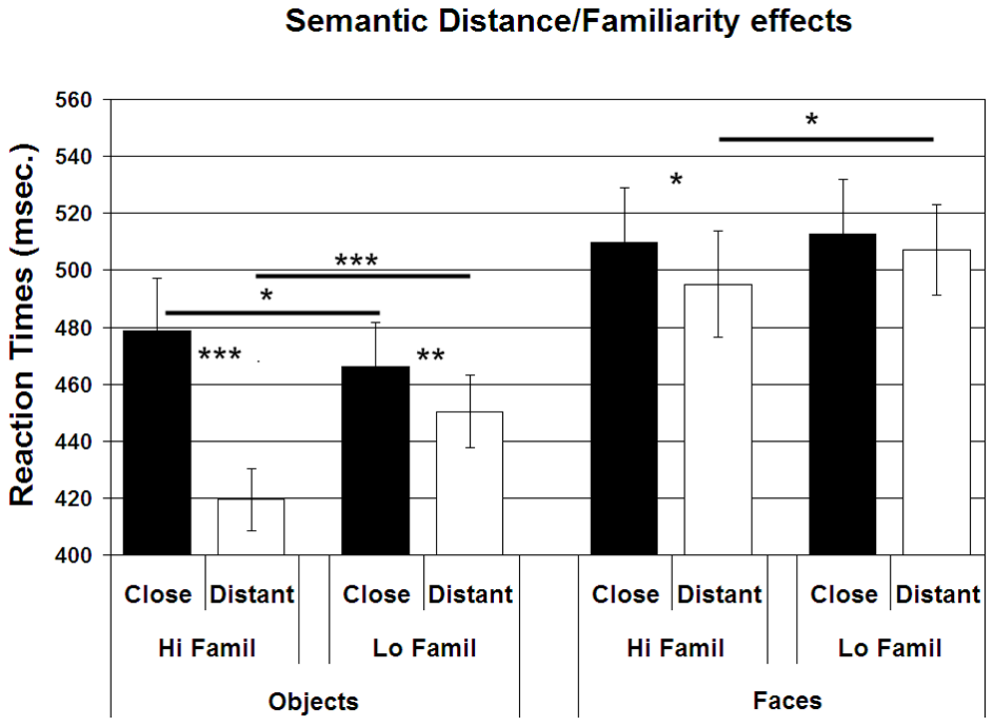
Effects of semantic variables. Effects of the manipulation of the semantic variables (semantic distance and familiarity) over the performance of the participants, regardless of TMS stimulation (category × distance × familiarity interaction). Vertical bars indicate Standard Error. *  = p<0.05; ** = p<0.01; *** = p<0.001.

Similarly to what was observed in accuracy, also the RTs analysis revealed main effects of category (F_(1,18)_ = 42.120; p<0.001; η^2^ = 0.701), with higher RTs for faces than objects, semantic distance (F_(1,18)_ = 29.384; p<0.001; η^2^ = 0.620) and familiarity (F_(1,18)_ = 12.159; p<0.001; η^2^ = 0.403). A significant interaction between category and semantic distance (F_(1,18)_ = 30.029; p<0.001; η^2^ = 0.625) was also found, with significant semantic distance effects for both categories (p<0.001 for Objects and p = 0.011 for Faces), but, as in accuracy, greater for objects (distant − close difference  = 37.55 ms) than for faces (distant − close difference  = 10.13 ms). At the level of RTs, however, the interaction between category and familiarity was not significant (F_(1,18)_ = 0.166; p = 0.689; η^2^ = 0.009). Moreover, significant distance × familiarity (F_(1,18)_ = 46.633; p<0.001; η^2^ = 0.721) as well as category × distance × familiarity (F_(1,18)_ = 11.129; p = 0.004; η^2^ = 0.382) interactions were found. Post hoc comparisons showed that (see Fig.3), while semantic distance effects were significant for both categories when the stimuli were highly familiar (all p<0.029), they disappeared with less familiar items for the category of faces (p = 0.327) but were still significant for the category of objects (p<0.001). On the other hand, for both categories subjects were slower in responding to lower familiarity items when they were unrelated (“distant” condition: p<0.001 for objects and p = 0.030 for faces). Conversely, when the items were semantically related (“close” condition) the familiarity effect disappeared for the category of faces (p = 0.606) and was actually reversed for the category of objects (p = 0.024), with subjects being slower in identifying highly familiar items.

More importantly however, a significant 3-way interaction between category, rTMS condition and semantic distance (F_(2,36)_ = 3.390; p = 0.045; η^2^ = 0.158) was found, suggesting a differential modulation of semantic distance effects for objects and faces induced by rTMS. In contrast, rTMS did not influence familiarity effects for the two categories (category × rTMS condition × familiarity interaction: F_(2,36)_ <1; p = 0.431; η^2^ = 0.046) and no differential modulation of rTMS was found on semantic distance effects for the two categories according to stimulus familiarity (category × rTMS condition × semantic distance × familiarity interaction: F_(2,36)_ <1).

**Figure 4:**
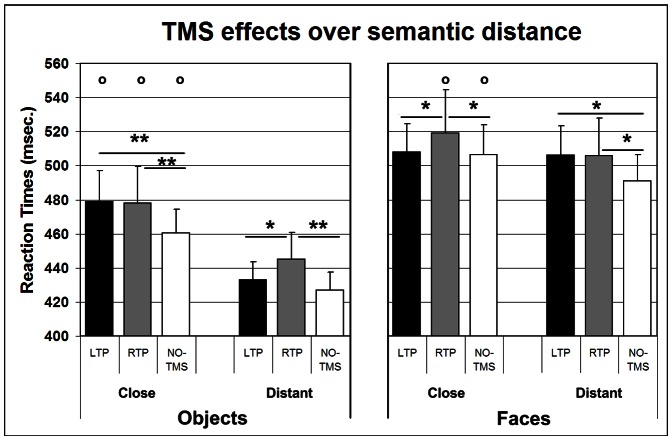
Effects of rTMS over the temporal poles for both categories. Effects of repetitive transcranial magnetic stimulation (rTMS) of the left (lTP) and right temporal poles (rTP) on Reaction times (RTs). After lTP stimulation there was a selective increment of RTs in the only close condition for Objects and in the only distant condition for Faces. Interferential effects after rTP stimulation were also found but were non specific for the semantic relation and tended to correlate with the visual similarity between the array stimuli. Vertical bars indicate Standard Error. * Asterisks indicate significant contrasts between rTMS conditions (*  = p<0.05; ** = p<0.01). **^O^** Indicates significant contrasts (p<0.05) between close and distant arrays of the same rTMS condition.

Post-hoc investigation of the source of the significant category × rTMS × semantic distance interaction showed that, for the category of objects, the stimulation of the lTP, as compared to the no-rTMS condition, induced a significant increment of RTs for the close (p = 0.004) but not for the distant object condition (p = 0.286), indicating an increase of semantic distance effects (see Fig. 4). The effect of semantic distance remained, however, largely significant in all the three rTMS conditions (p<0.001 for all close vs. distant comparisons). Stimulation of the rTP also induced a slowing of RTs for objects, but this was non specific for the semantic relation between the array stimuli and did not change the amount of semantic distance effects. Indeed, as compared to the no-rTMS condition, stimulation of rTP slowed RTs both in the close *and* in the distant conditions (p = 0.004 for both). As regards the direct confrontation between left and right TP stimulation, no difference was obtained between the two stimulation sites in the close condition (p = 0.870), but RTs in the distant condition were slower after rTP than lTP stimulation (p = 0.038).

Also for the category of faces there was a modulation of the semantic distance effect after the stimulation of lTP only. Indeed, while post hoc comparisons showed a significant semantic distance effect both in the no-rTMS (p = 0.015) and in the rTP conditions (p = 0.040; see Fig. 4), the effect of semantic distance completely disappeared after the stimulation of lTP (p = 0.790). This was due to a selective increase of RTs in the only “distant” condition, since subjects were slower in identifying unrelated faces after lTP rTMS than in baseline no-TMS condition (p = 0.015), but they showed no RTs increment when stimuli were closely related (p = 0.829). Similarly to the effects on objects, also for faces the stimulation of rTP induced a non-specific, generalized slowing of responses, since RTs increased in both the “close” (p = 0.039) and the “distant” (p = 0.012) conditions. As regards the direct comparison between left and right TP stimulation, no difference was obtained between the two stimulation sites in the distant condition (p = 0.969), but RTs in the close condition were marginally slower after rTP than lTP stimulation (p = 0.050).

It seems, therefore, that the stimulation of lTP and rTP led to very different effects on the performance of the participants. A modulation of the semantic distance effects in the absence of any influence on familiarity effects was found only after the stimulation of the lTP. On the contrary, the stimulation of the rTP seemed to induce a generalized slowing of responses which was not following any semantic dimension. Stimulation of the lTP, however, led to opposite semantic distance effect modulations for the two categories, namely a specific slowing of RTs for the close, but not distant, object condition and for the distant, but not close, face condition. Therefore, while the stimulation of lTP induced an *increase* in the semantic distance effect dimension for the category of objects, the stimulation of the same site *reduced* the size of the same effect for the category of faces.

### Influence of visual similarity

A common confound in the study of visually presented stimuli in semantic judgment tasks is the one concerning visual similarity. Indeed two (or more) objects belonging to the same semantic category or context tend to be also visually similar. Indeed, semantically close pairs were rated also more visually similar than distant pairs for both object (close = 4.011; sd = 2.256; distant = 1.951 sd = 1.076; Wilcoxon Matched-Pairs test: z = 3.724; p<0.001) and face (close = 3.382 sd = 1.231; distant = 2.620; sd = 1.042; Wilcoxon Matched-Pairs test: z = 3.724; p<0.001) categories. Close objects were, moreover, rated as more similar than close faces (Wilcoxon Matched-Pairs test: z = 2.984; p = 0.003), while the opposite was true for distant stimuli, with distant faces rated as more similar than distant objects (Wilcoxon Matched-Pairs test: z = 3.157; p = 0.002) (Bonferroni correction for all comparisons: p = 0.05/6 = 0.008).

The possibility therefore exists that any effect of *semantic* similarity might be confounded with, or influenced by, the *visual* similarity of the presented stimuli [48], [49]. In our participants, indeed, visual and semantic similarity judgments correlated highly with each other (r = 0.595 p<0.001). Thus, the rTMS effects might be due to perceptual blurring rather than to the alteration of semantic processing. To control for this alternative explanation of the described modulation of semantic distance after rTMS, we performed an item analysis using a partial correlation (Pearson coefficient) procedure. For each stimulus pair, we estimated the rTMS effects by calculating the ratio between the RTs after lTP and rTP stimulation and the corresponding RTs in the no-rTMS condition and we expressed them as percentage change of the no-rTMS. We then calculated the partial correlation between the lTP and rTP rTMS effects for each pair of stimuli and their visual similarity, once the influence of the semantic similarity between the stimuli is partialled out. The results showed that for the effects of lTP stimulation the influence of visual similarity was not significant (r = 0.114; p = 0.154), while for the right TP rTMS effects the influence of visual similarity was marginally significant (r = 0.155; p = 0.051).

While these results cannot exclude an influence of visual similarity in explaining the magnitude of the semantic distance effects, this influence seems to be clearly insufficient in explaining the effects of lTP stimulation according to the semantic distance between the stimuli. On the other hand, the positive correlations found between visual similarity and the effects of rTP stimulation might support the possibility that the RTs slowing found in this condition was more likely due to interference occurring at the perceptual level, with no influence of the semantic variables considered.

## Discussion

The characterization of person-specific semantic representations as a separate domain of knowledge, independent from the so-called general semantic knowledge, has been long debated and is still largely controversial from both the cognitive and anatomical points of view [5], [11], [13], [20], [21], [24], [50], [51]. The aim of the present study was to investigate both the cognitive and the anatomical underpinnings of person-specific knowledge as contrasted with the semantic knowledge for inanimate objects. In particular, from the cognitive point of view, we wanted to investigate whether the semantic representation of familiar people follows similar rules as that of common objects, being organized along dimensions of semantic distance (as suggested for example by Crutch and Warrington) [16] and familiarity. From the anatomical point of view, on the other hand, we aimed to investigate whether semantic information about familiar people involves the right or left anterior temporal regions and to assess whether this type of semantic knowledge is anatomically dissociable from that of common objects.

To these aims, we used low frequency rTMS over the lTP and rTP to interfere with semantic processing of common objects and famous faces in healthy individuals. We administered two speeded written word-to-picture matching tasks involving, respectively, object and face stimuli. In each task, a target concept had to be quickly recognized and selected from an array in which the target stimulus was paired with a distractor. The matching and distractor stimuli were varied in terms of semantic distance (close or distant) and familiarity (high or low) of their relative concepts. Both tasks were administered after rTMS of the lTP and rTP and in absence of any stimulation (no-rTMS). Ratings of visual similarity and semantic distance for each stimulus pair were collected from the same participants at the end of the testing session.

The results suggest that under condition of strict time pressure healthy participants committed a certain number of errors which, regardless of the category, were influenced by the semantic distance between the target and the distractor and by their familiarity. For both categories, the effects of lTP rTMS were modulated by the semantic distance, while rTP rTMS induced an overall slowing down of responses, in particular for stimuli with higher visual similarity.

### Cognitive organization of object and person specific knowledge

Results showed, in line with previous research (see Gainotti, 2007 for a review) [24], that recognizing familiar people was overall more difficult than recognizing common objects, probably because the knowledge of famous people is more variable and less consistent across individuals (see below). For both object and face stimulus categories, semantic distance and familiarity effects were reliably obtained in accuracy and RTs analyses, collapsing the rTMS conditions. However, the dimension of those effects differed across categories, with semantic distance effects larger for objects and familiarity effects larger for faces. This suggests that familiarity might play a more important role for the semantic organization of familiar people than it does for that of common objects, which seems to be more affected by semantic distance. Indeed, semantic distance effects remained significant in both high and low familiarity conditions for common objects. Conversely, they were significant only for highly familiar faces and were, instead, suppressed when faces were less familiar, likely reflecting the weakness of semantic representation of people that are less known. On the other hand, for both face and object categories, familiarity effects were strong for distant arrays, but were reduced or even reversed when stimuli were closely related, a condition which is commonly found in those patients who are affected by difficulties in accessing concepts [26], [27]. Overall, these results support the notion [32] that the category of familiar people, in keeping with that of common objects, is cognitively organized according to semantic distance criteria, the particular dimension manipulated in this study being occupation. According to our data it seems, thus, reasonable to suggest that both categories might share the same semantic organization principles. That familiarity seems to play a more important role for people than object knowledge may be probably due to a higher interpersonal variability in the degree of knowledge of famous people than of common objects.

### Anatomical underpinnings of objects and familiar people knowledge

The application of rTMS to the left anterior temporal regions did not induce overall impairments of the participants' performance in the object and face matching tasks, but rather caused an RTs slowing that clearly followed a semantic dimension. In particular, of the two semantic variables manipulated in the task, only semantic distance modulated the rTMS effects, while familiarity did not. Such effects of lTP rTMS again resemble those caused by brain damage in patients with difficulties in *accessing* semantic representations, since their performance is largely modulated by semantic distance but not by word frequency (or familiarity). In keeping with the interpretation of the semantic access dysfunctions in these patients [26], we suggest that rTMS might have induced disturbances in the connectivity among the nodes of the *semantic* network representing concepts that share many semantic attributes, are highly interconnected and are, thus, more prone to the spreading of rTMS interference with respect to unrelated concepts.

Our data, suggest moreover that left and right temporal poles might have a different role in concept representation, independently from their category. Indeed, the effects of lTP stimulation, for both semantic categories, were more consistently modulated by semantic (contextual) distance and were not influenced by perceptual variables such as the visual similarity between the stimuli. The contrary however was true after stimulation of the rTP, when RTs slowing down was obtained for both close and distant conditions, with no modulation of the semantic (contextual) distance effect, and it more reliably correlated with visual similarity. This would indicate that rTP rTMS might have affected the perception of the stimuli rather than their semantic representation. In sum, results suggest that the stimulation of *left* TP leads to *semantic* interference during object recognition, while the effects of the stimulation of *right* TP are more compatible with an interference occurring at a *perceptual* level.

The complimentary semantic and perceptual roles, respectively, of the left and right anterior temporal cortices might explain the mixture of perceptual and semantic deficits in recognizing familiar people [51], [52] shown by patients with dementia, who may have a bilateral progressive degeneration of the cortical regions of both temporal lobes. Furthermore, the left lateralization of the rTMS effects on semantic variables for both faces and objects and the right lateralization of the rTMS effects on perceptual variables are compatible with the results of Gorno-Tempini and colleagues [23]. In a PET study, the authors showed a left anterior temporal activation during identification of faces, but a right hemisphere lateralization for the *perceptual* analysis of faces. Importantly, the left anterior temporal area associated to the access to the personal information of faces partially overlapped with that associated to the access to object knowledge. In another PET study, Gorno-Tempini and Price [20] found that category specific activations in response to faces and buildings in the fusiform gyri were not modulated by the familiarity (fame) of the stimuli. Conversely, contrasting the brain activations in response to famous faces and buildings with that to non-famous stimuli revealed activations in the left anterior temporal regions.

The data by Gorno-Tempini and colleagues support the notion that the left anterior temporal cortex is involved in the semantic representation of concepts, independently of their category. Crucially, however, they did not manipulate the semantic distance between the concepts and, thus, did not test the involvement of the anterior temporal regions in the identification of entities at different levels of categorization. In the present study we manipulated the semantic distance between the target concepts and, thus, we could test the involvement of the left anterior temporal regions in the different levels of categorization required for their discrimination.

The present data do not fully support the view that the right and left anterior temporal lobes are involved in storing semantic representations in an “amodal” format (the so-called “Hub” account) [4]. Indeed, we found clear semantic effects only after the stimulation of the left temporal pole, while the stimulation of the right temporal pole induced interference more at a perceptual level. An alternative hypothesis on the differential roles of left and right temporal poles in the processing of semantic information is the one suggesting that the right temporal pole might process and store semantic information in a pictorial format while the left temporal pole might work more in a lexical-verbal format [53]. This hypothesis might be in keeping with the correlation between visual similarity and the interferential effects of the stimulation of the right temporal pole. In this view, visual similarity might be by all means a form of “semantic” dimension and the semantic distance “metric” might measure also the perceptual similarity between the exemplars of a category. This would explain why a “contextual” semantic metric of stimuli arrangement did not influence the performance when rTP was stimulated. Still, in the account that the right TP affected the semantic representation of objects and faces in a pictorial format, stimulation of rTP should have affected more the close pairs (visually more similar) and have again induced an increase of semantic distance effects. However, since our data showed that, for both categories, right TP stimulation induced an increment in RTs with both distant and close pairs, we do not have sufficient evidence to support this hypothesis and we have to favour a non-semantic (perceptual) account for the effect. However we cannot rule out that such perceptual effect might reflect a more general involvement of right TP in storing semantic representations in a pictorial format [53], [54], since our task was not *specifically* designed to investigate this issue. Indeed, patients with right temporal atrophy performed worse than those with a left temporal atrophy both on a face identification task and on a semantic task (the Pyramids and Palms Trees) presented in a pictorial format [5]. Future studies are needed to better investigate the perceptual or semantic nature of object and face representations in the right TP.

### Semantic representations in the left anterior temporal regions

Although lTP rTMS modulated semantic distance effects for both objects and faces, the RTs slowing down was selective for different semantic distance conditions in the two categories. Indeed, rTMS of lTP slowed RTs selectively for close items in the category of objects, but for distant ones in the category of faces. If this difference might seem surprising, it has to be kept in mind that the levels of semantic relatedness in the ‘close’ and ‘distant’ conditions may not correspond across categories. Indeed, the two categories of knowledge may be inherently different in terms of the level of categorization at which exemplars are recognized. It has been proposed [22] that faces are typically recognized at a subordinate (*individual*) level, since we are experts in this task that we perform very often and that is therefore highly automatic. In contrast, other classes of non-face objects are typically categorized at ‘basic’ levels, spanning from more general (e.g., ‘birds’ or ‘tools’) to more specific categories (e.g., ‘sparrow’ or ‘kitchen tools’). Thus, it is entirely plausible that the level of categorical organization of the close object condition (e.g., pairs of manipulable kitchen tools) corresponds to the basic level of categorization of the ‘distant’ face condition (e.g., pairs of famous persons from different occupational fields). This would explain why a selective and semantically driven effect of lTP rTMS has been found in our close object condition and distant face condition only. Possible support to this view might also come from the subjective ratings provided by the participants at the end of the experimental session (Figure 2). Indeed, the distant face pairs were rated more semantically associated than the distant object pairs, and their level of semantic distance tended to be therefore more similar to that of close object pairs. However, these data are to be interpreted with caution since participants rated the items that were more semantically distant and more familiar considering separately the object and face categories. Therefore, the items in the two categories may have been rated using different subjective judgement scales, whose levels were adapted according to the relative extent of semantic distance or familiarity within each category.

The fact that we did not observe any effect of lTP rTMS for the distant object condition, in which participants had to discriminate between, for example, a ‘kitchen’ and a ‘garage’ tool, might suggest that the lTP is not involved for general and superordinate levels of categorization. Rogers and colleagues [4] have, indeed, suggested that the representations encoded in the anterior temporal regions capture the degree of semantic relatedness among known concepts. Since closely related items, in contrast to unrelated ones, share similar patterns of activation in the anterior temporal regions [55], [56], interference in the activity of the anterior temporal regions induced by lTP rTMS might have caused a slowing of the participants' RTs for matching semantically related items only. Thus, the present results are in keeping with the role of the anterior temporal regions in storing semantic representations of concepts at specific levels of categorization [51], [57].

The absence of lTP rTMS effects in the *close face* condition, on the other hand, suggests that the stimulation of the lTP did not affect the access to the specific nodes representing the persons' identity independently from the semantic attributes (e.g., occupation) those persons share with other individuals. Indeed, since contextual information (occupation in this case) may be particularly important in driving the semantic representation of famous people, the discrimination between two individuals with the same occupation (e.g., two actors) requires the direct access to their identity nodes (e.g., Tom Cruise vs. Brad Pitt), that may not share relevant semantic attributes at such very specific (i.e., individual) level of categorization. A similar account has been proposed in a similar context by McNeil, Cipolotti and Warrington [58] to explain the selective preservation of the ability to access proper names in the case of a clear semantic access difficulty for common objects in a patient with a left fronto-temporal-parietal lesion. Indeed, it has been suggested that the crucial difference between proper and common nouns lies in the fact that proper names have a unique referent [59], [60], while common names refer to entities sharing an entire set of attributes (clustering them in the semantic space).

In sum, our data suggest that the role of the left anterior temporal regions in concept identification might be that of discriminating both object and face stimuli at specific levels of categorization, such as two different types of kitchen tools (i.e., our close object condition) or two different famous persons (i.e., our distant face condition). The same regions, instead, may not be involved in the recognition of stimuli at the superordinate levels of categorization that are sufficient to discriminate concepts belonging to different semantic contexts, such as ‘garage’ vs. ‘kitchen’ tools (i.e., our distant object condition) or public vs. personally familiar faces (not tested here). On the other hand, the same left anterior temporal regions may not even be involved in the recognition of stimuli at *very-specific* individual levels, such us discriminating two different exemplars of the same type of kitchen tool (e.g., different pots; not tested here) or two famous persons sharing the same occupational field (our close face condition). Future studies are needed to better understand the neural underpinnings of the fine-grained semantic discrimination required for such very specific, individual levels of categorization in which the identity node of the exemplar, either a face or an object, needs to be accessed [61]-[63].

## References

[1] Hodges JR, Patterson K, Oxbury S, Funnel E (1992) Semantic dementia. Progressive fluent aphasia with temporal lobe atrophy. Brain 115: 1783–1806. 1486461 10.1093/brain/115.6.1783

[2] Snowden J, Goulding P, Neary D (1989) Semantic dementia: a form of circumscribed cerebral atrophy. Behav Neurol 2: 167–182.

[3] Patterson K, Nestor PJ, Rogers TT (2007) Where do you know what you know? The representation of semantic knowledge in the human brain. Nat Rev Neurosci 8(12): 976–987. 18026167 10.1038/nrn2277

[4] Rogers TT, Lambon Ralph MA, Garrard P, Bozeat S, McClelland JL, Hodges JR, et al (2004) Structure and deterioration of semantic memory: a neuropsychological and computational investigation. Psychol Rev 111(1): 205–235. 14756594 10.1037/0033-295X.111.1.205

[5] Snowden JS, Thompson JC, Neary D (2004) Knowledge of famous faces and names in semantic dementia. Brain 127(Pt4): 860–872. 14985259 10.1093/brain/awh099

[6] Damasio H, Grabowski TJ, Tranel D, Hichwa RD, Damasio AR (1996) A neural basis for lexical retrieval. Nature 11 380(6574): 499–505. 10.1038/380499a0 8606767

[7] Evans JJ, Heggs AJ, Antoun N, Hodges JR (1995) Progressive prosopagnosia associated with selective right temporal lobe atrophy. A new syndrome? Brain 118 (Pt1): 1–13. 7894996 10.1093/brain/118.1.1

[8] Tyrrell PJ, Warrington EK, Frackowiak RS, Rossor MN (1990) Progressive degeneration of the right temporal lobe studied with positron emission tomography. J Neurol Neurosurg Psychiatry 53(12): 1046–1050. 2292695 10.1136/jnnp.53.12.1046 PMC488312

[9] Kitchener E, Hodges JR (1999) Impaired knowledge of famous people and events with intact autobiographical memory in a case of progressive right temporal lobe degeneration: implication for the organizazion of remote memory. Cog Neuropsychol 16(6): 589–607.

[10] Seidenberg M, Griffith R, Sabsevitz D, Moran M, Haltiner A, Bell B, et al (2002) Recognition and identification of famous faces in patients with unilateral temporal lobe epilepsy. Neuropsychologia 40(4): 446–456. 11684177 10.1016/s0028-3932(01)00096-3

[11] Thompson SA, Graham KS, Williams G, Patterson K, Kapur N, Hodges JR (2004) Dissociating person-specific from general semantic knowledge: roles of the left and right temporal lobes. Neuropsychologia 42(3): 359–370. 14670574 10.1016/j.neuropsychologia.2003.08.004

[12] Hodges JR, Graham KS (1998) A reversal of the temporal gradient for famous person knowledge in semantic dementia: implications for the neural organisation of long-term memory. Neuropsychologia 36(8): 803–825. 9751444 10.1016/s0028-3932(97)00126-7

[13] Miceli G, Capasso R, Daniele A, Esposito T, Magarelli M, Tomaiuolo F (2000) Selective deficit for people's names following left temporal damage: an impairment of domain-specific conceptual knowledge. Cog Neuropsychol 17(6): 489–516. 10.1080/02643290050110629 20945192

[14] Papagno C, Capitani E (2001) Slowly progressive aphasia: a four-year follow-up study. Neuropsychologia 39(7): 678–686. 11311298 10.1016/s0028-3932(01)00007-0

[15] Papagno C, Miracapillo C, Casarotti A, Romero Lauro LJ, Castellano A, Falini A, et al (2011) What is the role of the uncinate fasciculus? Surgical removal and proper name retrieval. Brain 134(Pt2): 405–414. 20959310 10.1093/brain/awq283

[16] Crutch SJ, Warrington EK (2004) The semantic organisation of proper nouns: the case of people and brand names. Neuropsychologia 42(5): 584–596. 14725797 10.1016/j.neuropsychologia.2003.10.009

[17] Douville K, Woodard JL, Seidenberg M, Miller SK, Leveroni CL, Nielson KA, et al (2005) Medial temporal lobe activity for recognition of recent and remote famous names: an event-related fMRI study. Neuropsychologia 43(5): 693–703. 15721182 10.1016/j.neuropsychologia.2004.09.005

[18] Leveroni CL, Seidenberg M, Mayer AR, Mead LA, Binder JR, Rao SM (2000) Neural systems underlying the recognition of familiar and newly learned faces. J Neurosci 15 20(2): 878–886. 10.1523/JNEUROSCI.20-02-00878.2000 PMC6772415 10632617

[19] Nakamura K, Kawashima R, Sato N, Nakamura A, Sugiura M, Kato T, et al (2000) Functional delineation of the human occipito-temporal areas related to face and scene processing. A PET study. Brain 123 (Pt9): 1903–1912. 10960054 10.1093/brain/123.9.1903

[20] Gorno-Tempini ML, Price CJ (2001) Identification of famous faces and buildings: a functional neuroimaging study of semantically unique items. Brain 124(Pt10): 2087–2097. 11571224 10.1093/brain/124.10.2087

[21] Kanwisher N, McDermott J, Chun MM (1997) The fusiform face area: a module in human extrastriate cortex specialized for face perception. J Neurosci 17(11): 4302–4311. 9151747 10.1523/JNEUROSCI.17-11-04302.1997 PMC6573547

[22] Gauthier I, Anderson AW, Tarr MJ, Skudlarski P, Gore JC (1997) Levels of categorization in visual recognition studied using functional magnetic resonance imaging. Curr Biol 7(9): 645–651. 9285718 10.1016/s0960-9822(06)00291-0

[23] Gorno-Tempini ML, Price CJ, Josephs O, Vandenberghe R, Cappa SF, Kapur N, et al (1998) The neural systems sustaining face and proper-name processing. Brain 121 (Pt11): 2103–2118. 9827770 10.1093/brain/121.11.2103

[24] Gainotti G (2007) Different patterns of famous people recognition disorders in patients with right and left anterior temporal lesions: a systematic review. Neuropsychologia 2007 45(8): 1591–1607. 10.1016/j.neuropsychologia.2006.12.013 17275042

[25] Warrington EK (1975) The selective impairment of semantic memory. Q J Exp Psychol 27: 635–657. 1197619 10.1080/14640747508400525

[26] Warrington EK, Cipolotti L (1996) Word comprehension. The distinction between refractory and storage impairments. Brain 119 (Pt2): 611–625. 8800952 10.1093/brain/119.2.611

[27] Warrington EK, Shallice T (1979) Semantic access dyslexia. Brain 102(1): 43–63. 427532 10.1093/brain/102.1.43

[28] Campanella F, Mondani M, Skrap M, Shallice T (2009) Semantic access dysphasia resulting from left temporal lobe tumours. Brain 132: 87–102. 19050031 10.1093/brain/awn302 PMC2638694

[29] Forde E, Humphreys G (1995) Refractory semantics in global aphasia: on semantic organisation and the access-storage distinction in neuropsychology. Memory 3(3–4): 265–307. 8574867 10.1080/09658219508253154

[30] Crutch SJ, Warrington EK (2003) Spatial coding of semantic information: knowledge of country and city names depends on their geographical proximity. Brain 126(Pt8): 1821–1829. 12821525 10.1093/brain/awg187

[31] Crutch SJ, Warrington EK (2004) A circumscribed refractory semantic access disorder: a verbal semantic impairment sparing visual semantics. Cog Neuropsychol 21: 299–315. 10.1080/02643290342000546 21038207

[32] Crutch S, Warrington EK (2005) Gradients of semantic relatedness and their contrasting explanations in refractory access and storage semantic impairments. Cog Neuropsychol 22(7): 851–876. 10.1080/02643290442000374 21038279

[33] Crutch SJ, Warrington EK (2005) Abstract and concrete concepts have structurally different representational frameworks. Brain 128(Pt3): 615–627. 15548554 10.1093/brain/awh349

[34] Jefferies E, Baker SS, Doran M, Lambon Ralph M (2007) Refractory effects in stroke aphasia: a consequence of poor semantic control. Neuropsychologia 45(5): 1065–1079. 17074373 10.1016/j.neuropsychologia.2006.09.009

[35] Pascual-Leone A, Bartres-Faz D, Keenan JP (1999) Transcranial magnetic stimulation: studying the brain-behaviour relationship by induction of ‘virtual lesions’. Phil Trans R Soc Lon B Biol Sci 1999 354(1387): 1229–1238. 10.1098/rstb.1999.0476 PMC1692644 10466148

[36] Brambati SM, Myers D, Wilson A, Rankin KP, Allison SC, Rosen HJ, et al (2006) The anatomy of category-specific object naming in neurodegenerative diseases. J Cog Neurosci 18(10): 1644–1653. 10.1162/jocn.2006.18.10.1644 17014369

[37] Campanella F, D′Agostini S, Skrap M, Shallice T (2010) Naming manipulable objects: anatomy of a category specific effect in left temporal tumours. Neuropsychologia 48(6): 1583–1597. 20144630 10.1016/j.neuropsychologia.2010.02.002

[38] Lubrano V, Filleron T, Demonet JF, Roux FE (2012) Anatomical correlates for category specific naming of objects and actions: A brain stimulation mapping study. Hum Brain Map. DOI: 10.1002/hbm.22189 10.1002/hbm.22189 PMC6869226 23015527

[39] Capitani E, Laiacona M, Mahon B, Caramazza A (2003) What are the facts of semantic category-specific deficits? A critical review of the clinical evidence. Cog Neuropsychol 20: 213–261. 10.1080/02643290244000266 20957571

[40] Gainotti G (2000) What the locus of brain lesion tells us about the nature of the cognitive defect underlying category-specific disorders: a review. Cortex 36(4): 539–559. 11059454 10.1016/s0010-9452(08)70537-9

[41] Wassermann EM (1998) Risk and safety of repetitive transcranial magnetic stimulation: report and suggested guidelines from the International Workshop on the Safety of Repetitive Transcranial Magnetic Stimulation, June 5–7, 1996. Electroencephalogr Clin Neurophysiol 108(1): 1–16. 9474057 10.1016/s0168-5597(97)00096-8

[42] Briggs GG, Nebes RD (1975) Patterns of hand preference in a student population. Cortex 11(3): 230–238. 1204363 10.1016/s0010-9452(75)80005-0

[43] Bertinetto P, Burani C, Laudanna A, Marconi L, Ratti D, Rolando C. Corpus e Lessico di Frequenza dell'Italiano Scritto (CoLFIS) http://www.istc.cnr.it/grouppage/databases.Accessed: 2013 April 22 Ref Type: Internet Communication

[44] Noble C (1954) The familiarity-frequency relationship. J Exp Psychol 47(1): 13–16. 13130804 10.1037/h0060025

[45] Smith R, Dixon T (1971) Frequency and the judged familiarity of meaningful words. J Exp Psychol 88: 279–281.

[46] Lambon Ralph MA, Pobric G, Jefferies E (2009) Conceptual knowledge is underpinned by the temporal pole bilaterally: convergent evidence from rTMS. Cer Cor 19(4): 832–838. 10.1093/cercor/bhn131 18678765

[47] Pobric G, Jefferies E, Ralph MA (2007) Anterior temporal lobes mediate semantic representation: mimicking semantic dementia by using rTMS in normal participants. Proc Natl Ac Sci USA 104(50): 20137–20141. 10.1073/pnas.0707383104 PMC2148435 18056637

[48] Gauthier I, James TW, Curby KM, Tarr MJ (2003) The influence of conceptual knowledge on visual discrimination. Cogn Neuropsychol 20(3): 507–523. 20957582 10.1080/02643290244000275

[49] Kerr NH, Winograd E (1982) Effects of contextual elaboration on face recognition. Mem Cognit 10(6): 603–609. 10.3758/bf03202443 7162420

[50] Gauthier I, Tarr MJ, Moylan J, Skudlarski P, Gore JC, Anderson AW (2000) The fusiform “face area” is part of a network that processes faces at the individual level. J Cogn Neurosci 12(3): 495–504. 10931774 10.1162/089892900562165

[51] Rogers TT, Hocking J, Noppeney U, Mechelli A, Gorno-Tempini ML, Patterson K, et al (2006) Anterior temporal cortex and semantic memory: reconciling findings from neuropsychology and functional imaging. Cogn Affect Behav Neurosci 6(3): 201–213. 17243356 10.3758/cabn.6.3.201

[52] Gauthier I, Skudlarski P, Gore JC, Anderson AW (2000) Expertise for cars and birds recruits brain areas involved in face recognition. Nat Neurosci 3(2): 191–197. 10649576 10.1038/72140

[53] Gainotti G (2012) The format of conceptual representations disrupted in semantic dementia: A position paper. Cortex 48(5): 521–529. 21807363 10.1016/j.cortex.2011.06.019

[54] Mion M, Patterson K, Acosta-Carbonero J, Pengas G, Izquierdo-Garcia D, Hong Y, et al. (2010) What the left and right anterior fusiform gyri tell us about semantic memory. Brain 133[11]: ,3256–3268. 10.1093/brain/awq272 20952377

[55] Hinton G, Shallice T (1991) Lesioning an attractor network: Investigations of acquired dyslexia. Psychol Rev 98(1): 74–95. 2006233 10.1037/0033-295x.98.1.74

[56] Plaut D, Shallice T (1993) Deep dyslexia: A case study of connectionist neuropsychology. Cog Neuropsychol 10(5): 377–500.

[57] Tyler LK, Stamatakis EA, Bright P, Acres K, Abdallah S, Rodd JM, et al (2004) Processing objects at different levels of specificity. J Cog Neurosci 16(3): 351–362. 10.1162/089892904322926692 15072671

[58] McNeil JE, Cipolotti L, Warrington EK (1994) The accessibility of proper names. Neuropsychologia 32(2): 193–208. 8190243 10.1016/0028-3932(94)90005-1

[59] Semenza C, Zettin M (1988) Generating proper names: A case of selective inability. Cog Neuropsychol 5(711): 721.

[60] Semenza C, Zettin M (1989) Evidence from aphasia for the role of proper names as pure referring expressions. Nature 7 342(6250): 678–679. 10.1038/342678a0 2480523

[61] Gross CG (2002) Genealogy of the “grandmother cell”. The Neuroscientist 8(5): 512–518. 12374433 10.1177/107385802237175

[62] Quian Quiroga R, Kreiman G, Koch C, Fried I (2008) Sparse but not “grandmother-cell” coding in the medial temporal lobe. Trends cog sci 12(3): 87–91. 10.1016/j.tics.2007.12.003 18262826

[63] Quiroga RQ, Reddy L, Kreiman G, Koch C, Fried I (2005) Invariant visual representation by single neurons in the human brain. Nature 435(7045): 1102–1107. 15973409 10.1038/nature03687

